# Autophagy and Breast Cancer: Connected in Growth, Progression, and Therapy

**DOI:** 10.3390/cells12081156

**Published:** 2023-04-14

**Authors:** Qitong Wu, Dipali Sharma

**Affiliations:** Department of Oncology, Johns Hopkins University School of Medicine and the Sidney Kimmel Comprehensive Cancer Center at Johns Hopkins, Baltimore, MD 21287-0013, USA

**Keywords:** breast cancer, autophagy, tumor dormancy, stemness, tamoxifen, trastuzumab, chemotherapy, autophagy inhibitors

## Abstract

Despite an increase in the incidence of breast cancer worldwide, overall prognosis has been consistently improving owing to the development of multiple targeted therapies and novel combination regimens including endocrine therapies, aromatase inhibitors, Her2-targeted therapies, and cdk4/6 inhibitors. Immunotherapy is also being actively examined for some breast cancer subtypes. This overall positive outlook is marred by the development of resistance or reduced efficacy of the drug combinations, but the underlying mechanisms are somewhat unclear. It is interesting to note that cancer cells quickly adapt and evade most therapies by activating autophagy, a catabolic process designed to recycle damaged cellular components and provide energy. In this review, we discuss the role of autophagy and autophagy-associated proteins in breast cancer growth, drug sensitivity, tumor dormancy, stemness, and recurrence. We further explore how autophagy intersects and reduces the efficacy of endocrine therapies, targeted therapies, radiotherapy, chemotherapies as well as immunotherapy via modulating various intermediate proteins, miRs, and lncRNAs. Lastly, the potential application of autophagy inhibitors and bioactive molecules to improve the anticancer effects of drugs by circumventing the cytoprotective autophagy is discussed.

## 1. Autophagy, a Complex Process Designed to Support Cell Death as Well as Survival via Recycling

Autophagy is a highly conserved process that functions to transport cargos to the lysosome for recycling and cellular degradation in eukaryotes [1]. Autophagy not only serves to remove defective or damaged organelles and cellular components by self-digestion, as a catabolic mechanism, it recycles substrates required to sustain homeostasis when nutrients are scarce [1,2]. The significance of proper autophagy extends to the soundness of immune cell function [3], intercellular communication [4], regulation of tissue-resident stem cells [5,6], and the integrity of the tissue barrier [7]. It can be triggered by tissue remodeling, long-term nutritional deprivation, quality control of organelles, cellular stress, and immune reaction [8]. In ideal circumstances, autophagy can be cytoprotective or destructive because an immoderate self-degradation process can be damaging [9]. As the key cellular process that regulates the stress response and thus takes part in the quality control in the cells [10,11], autophagy has a recondite impact on human lifespan and health [4]. Consequently, numerous human illnesses, including neurodegeneration, myopathies, cancer, aging, and lung, liver and heart diseases, as well as metabolic complications such as diabetes are linked to autophagic dysfunction [12].

Autophagy can be distinguished into four categories based on how the protein is transported to the lysosome [2]—microautophagy, chaperone-mediated autophagy, macroautophagy, and selective autophagy [2]. In microautophagy, the lysosomal membrane will undergo invagination or protrusion for cargo uptake [13]. Instead of manipulating membrane structures, chaperone-mediated autophagy uses chaperones for cargo identification that carry a specific pentapeptide motif. Subsequently, each of these components are then unfolded and individually translocated through the lysosomal membrane [14]. In contrast, macroautophagy generates double-membrane vehicles (autophagosomes) for cargo sequestration [15]. Guided by specific autophagy-related genes (ATGs) and BECN1 (Beclin-1), the initiation step of macroautophagy precedes phagophore elongation, autophagosome maturation, and fusion of lysosome and autophagosome. This process is concluded by proteolytic degradation of the cargo [2]. On the other hand, the macroautophagy of a particular cellular component is known as selective autophagy. Different from macroautophagy, the key regulator of selective autophagy is PINK1 (phosphatase and tensin homolog-induced putative kinase 1) [2]. The specificity of selective autophagy is preserved by ubiquitination or labeling of each cargo. In this process, p62 is an autophagy substrate that serves as a reporter [16]. Subsequently, autophagy receptors selectively bind to the tagged cargo and proceed to the formation of autophagosome [17,18]. These types of autophagy are mechanically varied, but they all culminate in lysosomal degradation of unwanted substances in the cell [1]. Adding to this complexity, several key proteins of autophagy machinery are known to be regulated by long non-coding RNAs (lncRNAs). LncRNA H19 promotes autophagy via modulating the Let-7–Lin28 axis [19], whereas LC3 and beclin1 are targeted by lncRNA ROR leading to autophagy promotion [20]. ATG10 is activated by direct binding of the lncRNA AGAP2-AS1–ELAVL1 complex to its promoter region [21]. LncRNAs, including HOTAIR, TALNEC2, EGOT, ZNF649, GAS5, DANCR, OTUD6B, and NAMPT, have been reported to regulate the expression of autophagy-associated proteins and impact cancer progression [22,23].

Interestingly, in cancer cells autophagy contributes to both death and survival [24]. The impact of autophagy in homeostasis serves to guard the genomic integrity of quiescent and growing cells in tissues [25]. Since genome instability is one of the cancer hallmarks, fidelity of autophagy has the leverage to prevent healthy cells from becoming cancerous [4,26]. It has been reported that autophagy in healthy cells prevents tumorigenesis via counteracting pro-oncogene stimuli [27]. Autophagy also activates the oncogene-induced senescence program, which keeps proliferative events at bay [28]. Nevertheless, many factors, such as the stage of disease, type of cancer, and condition of the patient can interfere with the real impact of autophagy in the progression of cancer [29].

## 2. Complex Relationship between Key Autophagic Proteins and Various Aspects of Breast Cancer Growth

Healthy cells commonly face intrinsic and extrinsic stress that can potentially result in genomic instability and mutations, which will aid neoplasia and hyperproliferation [30]. Autophagy serves to prevent such complications by eliminating tumorigenic stressors such as oncoproteins, protein aggregates, reactive oxygen species (ROS) production, and dysfunctional mitochondria [26,31,32,33,34,35,36,37,38,39]. Besides that, autophagy has roles in immune responses and inflammation [40,41,42,43]. As such, the maintenance of cellular integrity and defense against neoplastic transformation are both facilitated by autophagy [40]. Owing to the cytoprotective function of autophagy, it serves to suppress tumorigenesis in terms of cancer initiation. Indeed, an elevated gene signature for autophagy is observed in healthy mammary glands, which is found to be decreased as breast cancer progresses [44]. Indeed, autophagy is a complex multistep process involving multiple proteins that participate in breast cancer initiation, growth, and metastatic progression as well as recurrence.

### 2.1. BECN1 Negatively Associates with Breast Cancer

Macroautophagy is the most well-studied subtype of autophagy [9]. When cells are under stress, ULK1 is activated directly or indirectly leading to the recruitment of BECN1 and ATGs, thus allowing the assembly of molecular complexes, which subsequently lead to the initiation of phagophore formation [45,46]. BECN1 deficiency is observed in breast cancer [47]. Consistently, monoallelic loss of *BECN1* is often observed in human breast cancer cells [47,48]. Further, progression of ex vivo HER2-enriched breast tumor is hindered upon overexpression of *BECN1* [49]. In addition, monoallelic deletion of *Becn1* in FVB/N mice results in the development of mammary tumors after parity [50]. In fact, mammary tumorigenesis in MMTV-*Wnt1* mice with monoallelic *Becn1* deletion is more aggressive compared with those in mice with homozygous *Becn1* [50]. Among human breast cancer samples excluding HER2-enriched tumors, tumors with overexpression of WNT-related genes and a low mRNA level of BECN1 present a poorer prognosis, and they are primarily TNBC [50,51]. On the other hand, it has been recognized that the etiology and aggressive phenotype of TNBC is related to the activation of the Notch1 pathway [52]. Macroautophagy has roles in such oncogenic signaling as well. Of note, BECN1 can induce autophagic degradation of Notch1, which leads to a phenotype that diminishes Notch1-signaling-dependent tumorigenesis. Indeed, silencing *BECN1* in TNBC cell lines leads to an enhanced clonogenicity, migration, and anchorage-independent growth [53]. These studies suggest that the expression of the autophagic indicator, BECN1, hampers the progression of breast cancer.

### 2.2. Autophagy-Related Genes (ATGs) Have a Dual Impact on Breast Cancer

There are approximately twenty evolutionarily conserved ATGs that actively participate in the autophagic process. Depending on the context, some ATGs may contribute to the prevention of tumorigenesis [43]. For example, enrichment of ATG7 has a negative impact on growth and glycolysis in TNBC cells. Similarly, TNBC tumors that bear a higher level of ATG7 present better prognoses [54]. On the other hand, autophagy in human breast epithelial cells with mutationally active oncogenic Ras can be pro-tumorigenic [55]. In such mutated cells, an enhanced glycolysis capacity and proliferation is observed in autophagy-competent cells compared with autophagy-deficient cells. Additionally, more autophagy-competent cells undergo Ras-mediated adhesion-independent transformation, which suggest that autophagy has the potential to stimulate Ras-mediated tumorigenesis under certain metabolic conditions [55]. Of note, given that autophagy can stimulate Signal Transducer and Activator of Transcription 3 (STAT3), and that STAT3 is frequently activated in TNBC, modulation of autophagy influences the TNBC subtype the most [56].

### 2.3. FOXO Can Modulate Breast Cancer via Autophagy

As a putative tumor suppressor, Forkhead Box O (FOXO) is a transcription factor that takes part in regulating cellular homeostasis, the maintenance of stemness, and aging [43,57]. Downregulation of FOXO1 in breast cancer is related to a worse prognosis in breast cancer, especially in HER2-positive subtypes [58]. Similarly, nuclear localization of FOXO3 is found to be related to a reduced metastatic event in luminal-like breast cancer [59]. Interestingly, FOXO3 has the leverage to induce the expression of proteins that participate in the initiation and autophagosome formation in macroautophagy. In agreement with this, the loss of FOXO3 leads to diminished expression of several ATGs, resulting in a declined activity of autophagy [60,61,62]. Indeed, tumorigenesis can be stimulated by the absence of FOXO3, which implies suppression of FOXO3-mediated autophagy contributes to mammary carcinogenesis [60,61,62]. To add another level of complexity to this matter, FOXO3 also induces autophagy in cancer stem cells (CSCs) to preserve their well-being, and thus contributes to recurrence and metastasis [63,64]. Altogether, FOXO3 has the leverage to suppress tumorigenesis in healthy cells but may induce cytoprotective autophagy in cancer stem cells.

### 2.4. Autophagy Contributes to Reduced Drug Sensitivity in Breast Cancer

Breast-cancer-related mortality has been increasing in the past two decades [43,65]. Following preliminary diagnosis, metastatic relapse accounts for 90% of breast-cancer-related deaths, which is ascribed to the resurgence of dormant breast cancer cells [66,67]. Many therapeutics for breast cancer, namely chemotherapy, target actively dividing cells by destructing DNA and key proteins [68,69,70,71]. In such circumstances, autophagy serves to breakdown long-lived proteins, macromolecular waste, and damaged organelles. Residual cancer cells that survive the therapeutic assault may result in dormancy transformation. Since autophagy can be used as nutritional support, the cells will have time to repair and thus contribute to chemoresistance, relapse, and disease progression [72]. Autophagy thereby decreases drug sensitivity to breast cancer cells while protecting them. Hence, autophagy can be cytoprotective to breast cancer cells [72].

### 2.5. Autophagy Influences Tumor Dormancy in Breast Cancer

When the environment becomes unfavorable for growth, tumor cells can become quiescent, which is termed tumor dormancy [73]. It has been discussed that tumor dormancy largely contributes to metastasis, disease recurrence, and therapy resistance [73,74]. Dormant cancer cells can remain latent for decades before being activated to a proliferative state [75]. Autophagy not only supports the growth of dormant cells within the tumor microenvironment (TME) [76,77] but also participates in distant colonization and extravasation of dormant cells under environmental stress [66,78]. Upon inhibition of autophagy in vivo and in human preclinical models of dormant breast cancer cells with hydroxychloroquine (HCQ), a substantial decline of metastasis burden and cell viability have been observed [66]. This phenomenon is ascribed to the accumulation of damaged mitochondria and ROS, which in turns leads to cell death [72]. Further, 6-phosphofructo-2-kinase/fructose-2,6-biphosphatase 3 (PFKFB3) acts to promote cell cycle progression while inhibiting apoptosis [43]. PFKFB3 can also be a substrate for autophagosomal degradation by interacting with the autophagy receptor p62 [79]. Interestingly, metastatic breast cancer cells bear higher levels of PFKFB3 compared with dormant cells but have less autophagic activity. Of note, low levels of autophagy stabilize PFKFB3, which leads to activation of dormant cells to a metastatic state [79]. These findings suggest that autophagy promotes dormancy in breast cancer cells [43]. The AMP-activated protein kinase (AMPK) and mammalian target of rapamycin (mTOR) pathways are essential for autophagic regulation in tumor cells [80]. Canonically, activation of the mTOR complex 1 (mTORC1) directly phosphorylates and thus sequesters transcription factor EB (TFEB) in the cytoplasm [81,82]. Since TFEB is a chief transcriptional regulator of lysosomal and autophagy genes, activation of mTOR suppresses induction of autophagy at the transcriptional level [82]. For AMPK-mediated regulation of autophagy, AMPK is activated upon energy depletion, which in turn inhibits the autophagy regulatory complex, thus resulting in disruption of autophagosome biogenesis [83,84]. Additionally, activated AMPK can phosphorylate Unc-51-like kinase (ULK1) and the TSC1/TSC2 complex, thereby inducing autophagy via suppressing the activation of mTORC1 [80,85]. In breast cancer, environmental stress stimulates the secretion of auto- and paracrine signaling factors, which block phosphoinositide 3-kinase (PI3K) activation and lead to the inactivation of AKT and mTOR, thereby resulting in the activation of autophagy [43,75]. The PI3K/AKT/mTOR pathways can be inhibited by Diras Family GTPase 3 (DIRAS3), which is found to be enriched in dormant breast cancer cells [75,86,87], suggesting that autophagy may contribute to tumor dormancy in breast cancer and thus plays a role in chemoresistance.

### 2.6. Autophagy Influences Hypoxia, Chemoresistance, and Stem-like Phenotype in Breast Cancer

It is known that cancer stem cells are a significant contributor to the development of chemoresistance in breast cancer [88,89,90,91]. Autophagy enables the survival of CSCs under hypoxia in the tumor microenvironment [92]. In fact, a subpopulation of TNBC cancer stem cells stay in an autophagic state in relation to hypoxia [93]. Indeed, environmental stress such as nutrient deprivation and hypoxia can lead to the activation of autophagy for cellular component recycling in order to sustain survival [94,95,96]. In cancer cells, hypoxia-inducible factor-1 (HIF-1) is the main regulator of hypoxic conditions [97,98]. Upon activation of HIF-1, stemness can be triggered via several pathways such as activation of NANOG, SOX2, SOX17, etc. [98,99,100]. Importantly, hypoxia stimulates autophagy via HIF-1α [101], a subunit of HIF-1. HIF-1α is involved in the generation, differentiation, invasion, plasticity, and therapeutic resistance of CSCs [92]. In two stem-like breast cancer cell lines, induction of stemness can be performed by autophagy via the EGFR/Stat3 and TGFβ/Smad pathways in a murine model [102]. It is also reported that inhibition of autophagy in certain breast cancer cell lines results in a decreased stemness phenotype [56,103]. Other than that, dormant stem-cell-like breast cancer cells express autophagy markers, and upon inhibition of autophagy using 3-methyladenine (3-MA), these cells are transformed to active state [104]. Moreover, doxycycline not only inhibits EMT (epithelial–mesenchymal transition) and stemness markers in breast cancer stem cells but also causes a down-regulation of autophagy activity [105], suggesting the possibility that autophagy may play a role in stemness [106].

In patient-derived xenografts, suppression of autophagy via inhibition of BECN1 leads to re-sensitization of chemoresistant cells to therapy [93], which emphasizes the role of autophagy in the development of chemoresistance. In luminal and HER2-enriched subtypes of breast cancer, similar results are observed, as chemoresistant cells not only have an elevated autophagic activity compared with their drug-sensitive counterparts but inhibition of autophagy also results in the restoration of chemosensitivity [107,108,109,110]. Further, expression of mesenchymal markers, vimentin, and the stem cell marker CD44 is increased upon autophagic activity in CSCs [106]. Additionally, self-renewal of a hormone-independent murine breast cancer cell line LM38-LP requires autophagy [111]. Interestingly, disruption of circadian rhythm is related to the acquirement of chemoresistance [112]. By itself, melatonin suppresses the development of chemoresistance in breast cancer by interfering with tumor metabolism [112,113]. However, the combination of dim light at night (dLAN) results in the activation of STAT3, which is often overexpressed in paclitaxel-resistant breast cancer [112]. Under the synergetic influence of dLAN and melatonin, the activated STAT3 inhibits DIRAS3 in an epigenetic manner, resulting in decreased autophagic activity and increased resistance of breast cancer to paclitaxel [112]. This finding implies that DIRAS3 can be a regulator for the development of chemoresistance via autophagy. On the other hand, it has been demonstrated that chemotherapeutics can trigger autophagy, which enhances the survival of CSCs [114]. In TNBC, inhibiting autophagy with chloroquine (CQ) causes the accumulation of dysfunctional mitochondria and ROS in CSCs, which results in cell death [115]. Interference of autophagy also disrupts the preservation of breast CSCs. When combining autophagy inhibitors and chemotherapy, a decreased expression of stemness markers is observed along with an increased sensitivity to chemotherapeutics and a decreased cancer cell viability and metastasis [105,111,115,116]. Indeed, autophagy can be cytoprotective by contributing to induced chemosensitivity in breast cancer cells [72]. 

### 2.7. Intermediate Steps in the Autophagic Process Play an Important Role in Breast Cancer

Autophagy flux can impact how breast cancer cells respond to treatment. Unsurprisingly, in-depth inquiries about autophagy have discovered that intermediate regulation of autophagy flux can impact the influence of autophagy on therapeutic resistance [72]. For example, the reporter in selective autophagy, p62, also has roles in the proteolytic system [16]. It not only functions to deliver ubiquitinated proteins to the proteasome for breakdown but also governs protein quality by binding with ubiquitinated cargoes while shuttling between the nucleus and cytoplasm [16]. Upon administration of bortezomib (a proteasome inhibitor), an elevated p62 expression is observed, which implies the failure in the turnover of autolysosomal protein, demonstrating the interplay between proteasomal degradation and selective autophagy [117]. Breast cancer cells fail to restore metabolic homeostasis via autophagy upon inhibition of autophagosomal degradation using obatoclax [118]. Further, the combined treatment of bortezomib and obatoclax in antiestrogen-resistant breast cancer cells results in a hindered autolysosomal function without preventing the formation of autophagosomes [117,118]. Interestingly, this sensitizes antiestrogen-resistant breast cancer cells to tamoxifen [76,118], which indicates that the indirect influence of the proteasome pathway can contribute to drug resistance. On the other hand, lysosomal-associated protein transmembrane 4β (LAPTM4B) is an essential maturation step in autophagy but also plays a significant role in lysosomal activities [119]. Expression of LAPTM4B is positively related to chemoresistance in breast cancer [119]. From the mechanistic standpoint, deficiency of LAPTM4B leads to an enhanced permeability of the lysosomal and the autolysosomal membranes [120]. Consequently, drugs can enter the nucleus more readily. The increased permeability of cathepsin also causes cathepsin to be released, which triggers lysosomal-mediated programmed cell death [120]. LAPTM4B deficiency significantly hinders the fusion of lysosomes and autophagic bodies, which begets the accumulation of autophagosomes, resulting in cell death [121] and thus diminishing therapeutic resistance. In summary, simply increasing or decreasing autophagic activity is not a wise route to regulate autophagy-mediated therapeutic resistance in breast cancer. It is also important to inquire how autophagy regulates the sensitivity of breast cancer to therapeutics [72].

## 3. A multifaceted Involvement of Autophagic Processes Modulate the Efficacy of Breast Cancer Therapeutics

Several studies suggest the involvement of autophagy in endocrine therapy, chemotherapy as well as immunotherapy. As autophagy can be cytoprotective as well as cytotoxic, its impact on various therapies can be context dependent, leading to either the development of resistance or increased efficacy [122,123,124,125].

### 3.1. Autophagy and Endocrine Therapy—Response and Resistance

**Figure 1:**
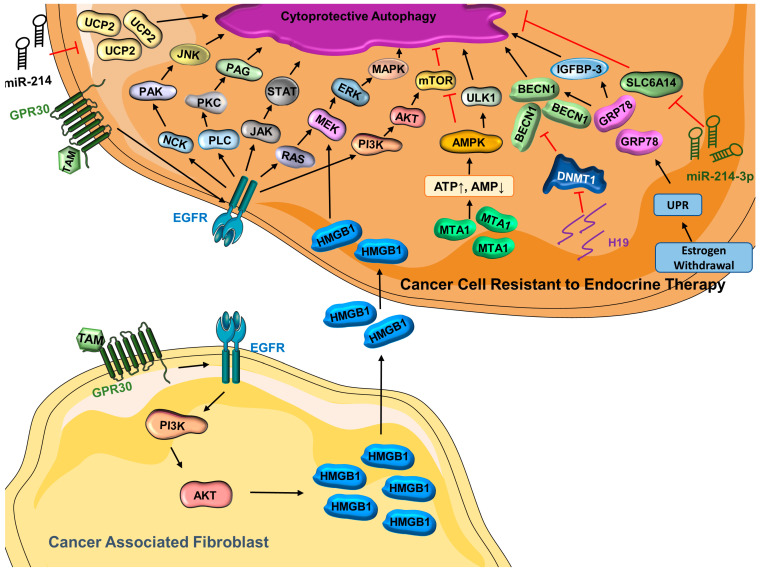
**Cytoprotective autophagy is activated in cancer cells resistant to endocrine therapy** (ATP = adenosine triphosphate; AKT = protein kinase B; AMP = adenosine monophosphate; AMPK = AMP-activated protein kinase; BECN1 = Beclin-1; DNMT1 = DNA-methyltransferase 1; EGFR = epidermal growth factor receptor; ERK = extracellular signal-regulated kinase; GRP78 = glucose-regulated protein 78; GPR30 = G protein coupled estrogen receptor; H19 = long noncoding RNA H19; HMGB1 = high mobility group box 1; IGFBP-3 = insulin-like growth factor binding protein 3; JAK = Janus kinase; JNK = c-Jun N-terminal kinase; MAPK = mitogen-activated protein kinase; MEK = mitogen-activated protein kinase kinase; miR-214 = microRNA 214; miR-214-3p = microRNA 214-3p; MTA1 = metastasis-associated antigen 1; mTOR = mammalian target of rapamycin; NCK = non-catalytic region of tyrosine kinase; PAG = glycosphingolipid-enriched microdomains; PAK = p-21-activated kinase; PI3K = phosphoinositide 3-kinase; PKC = protein kinase C; PLC = phosphoinositide-specific phospholipase C; SLC6A14 (solute carrier family 6 member 14); STAT = signal transducer and activators of transcription; TAM = tamoxifen; UCP2 = mitochondrial uncoupling protein 2; ULK1 = Unc-51 like autophagy activating kinase 1; UPR = unfolded protein response).

Estrogen-receptor-positive breast cancer accounts for the majority of breast cancer cases, and they are predominantly treated with endocrine therapy [126]. The estrogen receptor antagonist tamoxifen has been successfully used for over 40 years [127]; however, some cancers do not respond to tamoxifen [128], owing to intrinsic or acquired resistance [129]. Several mechanisms associated with tamoxifen resistance including alteration of the estrogen receptor 1 (*ESR1*) gene, modulation of coregulator proteins, and modification of proliferative signaling pathways have been put forth [129,130,131]. Recent studies have explored the role of autophagy in tamoxifen resistance [132,133]. G protein coupled estrogen receptor (GPR30), a transmembrane estrogen receptor, contributes to tamoxifen resistance [134] via transactivating epidermal growth factor receptor (EGFR) [134,135,136], leading to the activation of several pathways that regulate autophagy, including the NCK/PAK/JNK, JAK/STAT, PI3K/AKT/mTOR, PLC/PAG/PKC, and MAPK pathways [137,138]. GPR30 is highly expressed in breast-cancer-associated fibroblasts [139,140], and GPR30-induced gene activation results in an enrichment of high mobility group box 1 (HMGB1) [132], which induces tamoxifen resistance through the MEK–ERK signaling pathway while increasing autophagic activities. A higher level of MTA1 (metastasis associated antigen 1) and autophagic activity has been observed in tamoxifen-resistant cells compared with their non-resistant counterparts [141]. Interestingly, knocking down MTA1 in tamoxifen-resistant cells not only sensitizes them to tamoxifen but also decreases autophagic activity [141,142]. In fact, the tamoxifen resistance conferred by MTA1 is associated with modification of autophagic activity [141]. Mechanistically, as evidenced by an increased AMP:ATP ratio, stable expression of MTA1 leads to the activation of AMPK and increased availability of p-PRKAA (phospho-protein kinase AMPK-activated catalytic subunit alpha), which increases autophagy in tamoxifen-resistant cells [141]. Interestingly, both autophagic activity and tamoxifen resistance were substantially decreased when PRKAA is knocked down in MCF7/TAMR-1 cells, even when MTA1 is overexpressed, implying that the AMPK pathway is essential to regulate autophagy, which is important for tamoxifen resistance [141]. When it comes to endocrine resistance, estrogen withdrawal can activate UPR (unfolded protein response) [143], leading to the release of glucose-regulated protein 78 (GRP78) [144], which balances pro-survival autophagy and pro-death apoptosis while bestowing endocrine resistance in ER-positive breast cancer [145,146]. Knockdown of GRP78 in MCF7/LCC9 (resistant to fulvestrant and tamoxifen) and MCF7-RR (tamoxifen-resistant) results in re-sensitization to the respective therapeutics [145]. Mechanistically, GRP78-mediated signaling may suppress apoptosis and stimulate autophagy to help cells endure the stress [147] via inhibition of mTOR [145,148]. In the breast tumor microenvironment, it has been reported that GRP78 also promotes autophagy by binding to insulin-like growth factor binding protein-3 (IGFBP-3), which is associated with poor prognosis [149]. Additionally, GRP78 indirectly activates BECN1 to promote autophagy in breast cancer cell lines (Figure 1) [148,150,151].

In addition to the genes/proteins associated with tamoxifen resistance and autophagy, recent studies have pointed out that long noncoding RNAs (lncRNAs) are involved in tamoxifen resistance [133,152,153]. In line with this, lncRNA H19 is found to be highly enriched in breast cancer cell lines and tumor tissues that are resistant to tamoxifen, and H19 knockdown in tamoxifen-resistant models results in a resensitization to tamoxifen [133]. Importantly, the expression of H19 is positively related to autophagic activity in MCF7/TAMR cells. Increased methylation in the promoter region of BECN1 is observed upon H19 silencing in tamoxifen-resistant cells. It is evident that H19 promotes autophagy via epigenetic modification of BECN1 [133]. As another type of non-coding molecule, microRNAs (miRs) also regulate a number of cellular processes including autophagy [72]. Endocrine resistance is negatively related to the expression of miR-214, which directly targets uncoupling protein 2 (UCP2) [154]. Interestingly, while it is well-recognized that the activation of mTOR leads to the inhibition of autophagy [82,155,156], UCP2 overexpression contradictorily leads to the enhanced activity of both the PI3K–Akt–mTOR signaling pathway and autophagic activity [154]. A previous study has speculated that the influence of UCP2 in autophagy and endocrine resistance is through the regulation of the PI3K–Akt–mTOR signaling pathway [154,157]. However, more research is needed to better understand the miR-214–UCP2-mediated autophagy and tamoxifen resistance in breast cancer [154]. Similarly, miR-23b-3p inhibits SLC6A14 (Solute Carrier Family 6 Member 14), a basic amino acid transporter, and results in an altered level of amino acids [158]. Such disruption may stimulate autophagy, and it has been reported that inhibition of SLC6A14 enhances autophagic activity in colon cancer [159,160]. Consistent with this, it has been reported that tumors resistant to endocrine therapy have a reduced SLC6A14 activity and upregulation of miR-23b-3p, leading to overactivation of cytoprotective autophagy that contributes to the development of therapeutic resistance [158]. Collectively, these findings reveal the importance of autophagy in the development of endocrine resistance in breast cancer (Figure 1).

### 3.2. Autophagy and Targeted Therapy—Response and Resistance

**Figure 2:**
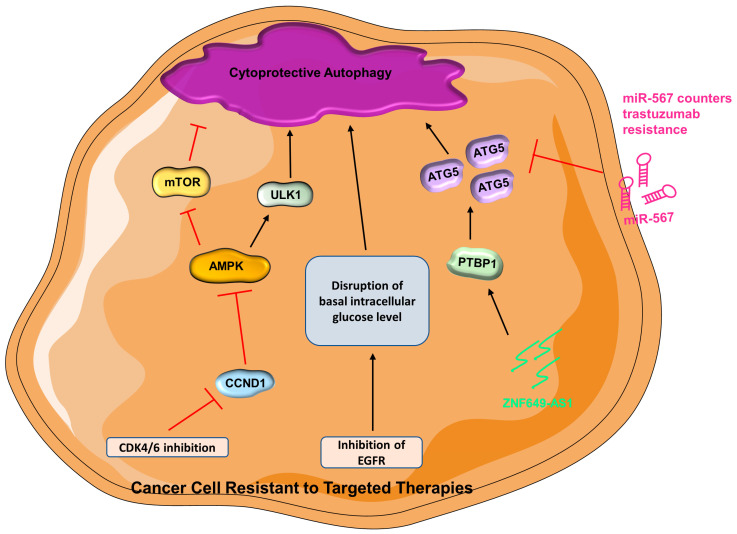
**In cancer cells resistant to targeted therapies, cytoprotective autophagy is promoted** (AMPK = AMP-activated protein kinase; ATG5 = autophagy-related gene-5; CCND1 = cyclin D1; CDK4/6 = cyclin-dependent kinase 4 and 6; EGFR = epidermal growth factor receptor; miR-567 = microRNA 567; mTOR = mammalian target of rapamycin; ULK1 = Unc-51 like autophagy activating kinase 1; PTBP1 = polypyrimidine tract binding protein 1; ZNF649-AS1 = ZNF649 Antisense RNA 1).

***Trastuzumab (Herceptin)***—The Her2-positive breast cancer subtype accounts for ~20% of all breast cancers [161], and it is associated with poor prognosis [162]. Trastuzumab is a monoclonal antibody that targets the extracellular region of HER2 serving as a targeted therapy for Her2-positive breast cancer; however, many patients develop resistance to trastuzumab following an initial response [163]. Multiple modulators of trastuzumab resistance have been uncovered. Sphingosine kinase 1 is a protooncogene associated with therapeutic resistance in breast cancer [164,165] and can be inhibited with the synthetic structural analog of sphingosine, Fingolimod (FTY720) [166,167]. Interestingly, FTY720 inhibits autophagy and triggers apoptosis in trastuzumab-resistant breast cancer cells [168]. In fact, co-treatment of FTY720 and trastuzumab results in a substantial growth inhibition compared with monotherapy [168], and the results are comparable to autophagy inhibitors [168,169], implying that FTY720 restrains autophagy to reverse therapeutic resistance while activating apoptosis [168,170]. Autophagy can be regulated by miRNA via posttranscriptional regulation [72,171]. As an important regulator of many biological processes, miRNAs have been demonstrated to participate in tumor progression and treatment response [172]. Unsurprisingly, miRNA can regulate autophagy via posttranscriptional modulation of autophagy-related protein expression [173]. Interestingly, miR-567 promotes the efficacy of trastuzumab in trastuzumab-resistant breast cancer cells by targeting ATG5, which results in the suppression of autophagy [173]. An enhanced expression of a lncRNA, ZNF649-AS1 (ZNF649 Antisense RNA 1), is detected in trastuzumab-resistant breast cells compared with trastuzumab-sensitive cells [174]. Inhibition of ZNF649-AS1 in trastuzumab-resistant cells results in suppression of autophagy and cells are re-sensitized to trastuzumab [174]. Further investigation presents that ZNF649-AS1 binds to polypyrimidine tract binding protein 1 (PTBP1), which stimulates the translation of ATG5 and thus results in enhanced autophagic activity, thereby contributing to the acquisition of trastuzumab resistance (Figure 2) [174].

***EGFR inhibitors***—As an important therapeutic target in cancer, the epidermal growth factor receptor (EGFR) family is often abnormally activated in many types of cancers and plays an important role in their development and progression [175,176]. Lapatinib is a small molecule tyrosine kinase inhibitor of HER2 and EGFR [177]. Mechanically, lapatinib binds to the cytoplasmic ATP-binding sites of HER2 and EGFR, thus preventing the binding of ATP and resulting in the inhibition of tyrosine kinase phosphorylation and downstream events [177]. Lapatinib-resistant breast cancer cells have higher levels of autophagic activity and they can be sensitized to treatment upon inhibition of autophagy [178]. Lapatinib-induced autophagy requires p62, which can be inhibited by a triterpenoid extracted from Schisandra plants (P3–15) to resensitize TNBC to lapatinib treatment in vitro and in vivo [179]. It is clear that autophagy aids in the development of lapatinib resistance, but the underlying mechanisms are yet to be elucidated [178]. Similar to lapatinib, gefitinib is also a tyrosine kinase inhibitor that targets EGFR and HER2 in breast cancer [180,181,182,183]. Among TNBC patients, 40% of them have enrichment of EGFR [184,185], but inhibiting EFGR is generally not efficient for the treatment of TNBC patients owing to drug resistance. Increasing evidence has pointed to the contribution of autophagy in resistance to EGFR inhibitors [186]. Interestingly, TNBC is sensitized to gefitinib therapy when autophagy is suppressed, which implies that the combination of autophagy inhibitor and gefitinib can be a good therapeutic strategy in treating TNBC patients who have enriched EGFR [187]. Silencing EGFR in SKBR3 (HER2^+^ breast cancer cell line) and MCF7 cells results in an elevated autophagy response. It is speculated that inhibition of EGFR in both gefitinib-sensitive or -insensitive cell lines can lead to disruption of basal intracellular glucose levels via inhibition of tyrosine kinase, and thus autophagy is triggered to offset the energy depletion [188,189]. Additionally, gefitinib-mediated autophagy activation can be a consequence of altered downstream signaling of EGFR and HER2 [188]. Yet again, more studies are needed to explore how autophagy contributes to therapeutic resistance.

***CDK4/6 inhibitors***—Cyclin-dependent kinases are a group of proteins that govern the cell cycle progression [190]. Cyclin-dependent kinase 4 (CDK4) and CDK6 are dysregulated in cancer cells and many preclinical studies have demonstrated the hyperactivity of the cyclin D–CDK4/6 axis, rendering CDK inhibitors as desirable therapeutic approaches [191,192,193]. CDK4/6 activation results in the phosphorylation of Serine/threonine kinase 11 (STK11) at S325 by activated cyclin D1 (CCND1, a regulatory subunit of CDK4/6). Activation of CDK4/6 leads to inactivation of AMPK, which in turn relieves its suppression on mTOR; activated mTOR can then lead to inhibition of autophagy [194,195]. The current approved CDK4/6 inhibitors are Abemaciclib, Palbociclib, and Ribociclib [196,197]. It is recognized that CDK4/6 inhibitors promote autophagy in various cancer models, including breast cancer [198,199,200]. Mechanistically, CDK4/6 inhibition renders CCND1 inactive, leading to AMPK activation and increased autophagy [194,201]. Indeed, when autophagy is inhibited, the therapeutic response of breast cancer to palbociclib is greatly increased [200], which suggests that inhibition of autophagy can be an attractive avenue for overcoming drug resistance [200,202]. Consistently, transcriptomic profiling results of palbociclib-sensitive and -resistant breast cancer cells reveal that resistant cells have upregulation of many autophagy-related genes [203]. Additionally, autophagy assists cells in managing stress from CDK4/6 inhibition by preventing apoptosis [203]. Combining CDK4/6 inhibitors and lysosomal destabilizers results in improved therapeutic efficacy in CDK4/6-inhibitor-resistant TNBC cells [204]. This demonstrates that lysosomes can be a therapeutic target to cope with resistance to CDK4/6 inhibitors (Figure 2) [205].

### 3.3. Autophagy and Radiotherapy—Response and Resistance

**Figure 3:**
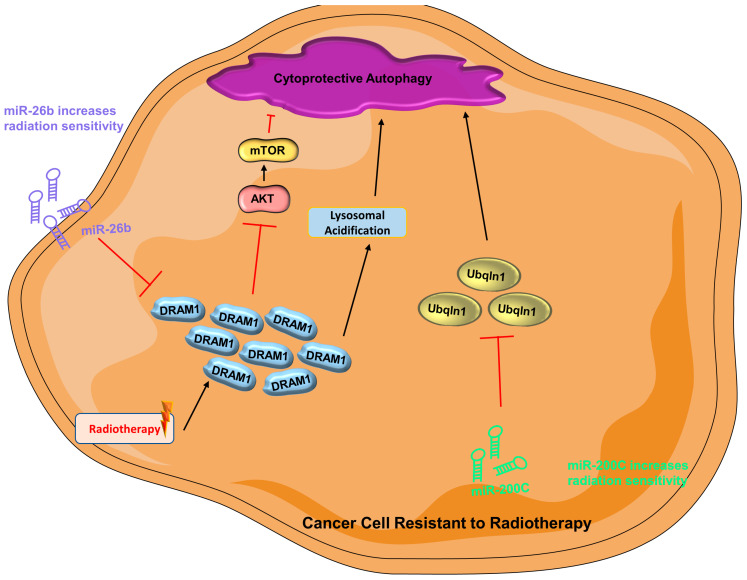
**Cytoprotective autophagy is activated in cancer cells resistant to radiotherapy** (AKT = protein kinase B; DRAM1 = damaged-regulated autophagy modulator 1; miR-200C = microRNA 200c; miR-26b = microRNA 26b; mTOR = mammalian target of rapamycin; PI3K = phosphoinositide 3-kinase; Ubqln1 = Ubiquilin-1).

In radiotherapy, cancer cells are subjected to high physical energy of radiation that causes enormous DNA damage resulting in cell death [206]. Autophagy may be stimulated to govern cancer cell survival post-radiotherapy [207]. Damaged-regulated autophagy modulator 1 (DRAM1) encourages autophagy, possibly by suppression of the AKT signaling pathway or augmentation of lysosomal acidification [208]. Unsurprisingly, miRNA may regulate the effect of radiotherapy via modulation of autophagy. As reported by a recent study, overexpressing miR-26b can lead to downregulation of DRAM1, which in turn leads to decreased autophagic activity and increased radiation sensitivity in breast cancer cells [209]. Similarly, miR-200C has been demonstrated to downregulate Ubqln1 (ubiquitin 1) when expressed ectopically in breast cancer [210]. In autophagy-mediated degradation, Ubqln1 is important in autophagosome maturation [211]. In a combined clinical and in vitro study, expression of miR-200C results in enhanced sensitivity to radiotherapy, which is ascribed to downregulation of Ubqln1 and thus reduction of autophagy (Figure 3) [210].

### 3.4. Autophagy and Chemotherapy—Response and Resistance

**Figure 4:**
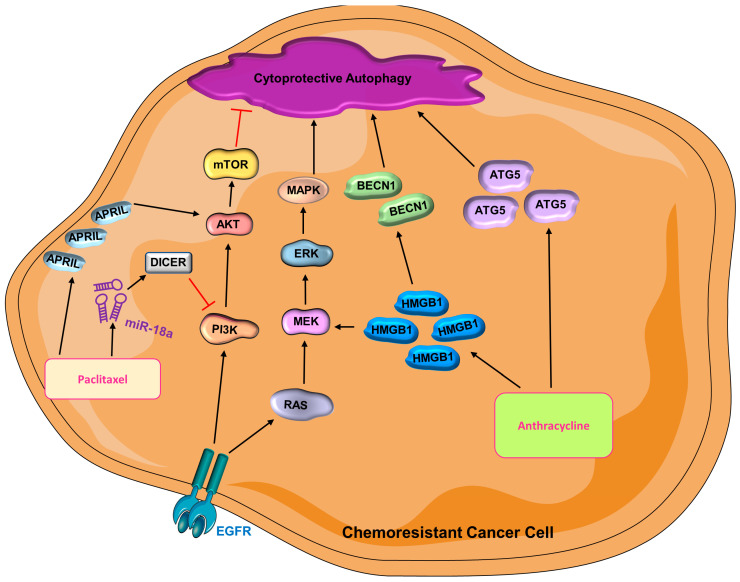
**Cytoprotective autophagy is activated in chemoresistant cancer cells** (AKT = protein kinase B; BECN1 = Beclin-1; ATG5 = autophagy-related gene-5; EGFR = epidermal growth factor receptor; ERK = extracellular signal-regulated kinase; HMGB1 = high mobility group box 1; MAPK = mitogen-activated protein kinase; MEK = mitogen-activated protein kinase kinase; miR-18a = microRNA 18a; mTOR = mammalian target of rapamycin; PI3K = phosphoinositide 3-kinase).

***Anthracycline***—Doxorubicin and epirubicin are both anthracyclines that are widely used chemotherapeutic agents that interfere with DNA replication and transcription, thus stopping cell proliferation [212]. As a member of the high mobility group protein superfamily, HMGB1 has secretory and intracellular activity and participates in breast cancer tumorigenesis [210,213,214]. The role of HMGB1 in the cytoplasm is associated with BECN1-mediated autophagy [215], metastasis, and chemo- and radiotherapy resistance in breast cancer [216,217,218,219,220]. Its contribution to therapeutic resistance is ascribed to the induction of autophagy [221,222,223]. The therapeutic efficacy of doxorubicin can be improved by inhibiting Med19 (Mediator Complex Subunit 19), which downregulates HMGB1 and results in the suppression of autophagy [219]. HMGB1 is positively related to autophagy level, NFκB/p65 activity, and doxorubicin-resistance in breast cancer cells [219]. The silencing of HMGB1 also reduces radiotherapy resistance in breast cancer, which is known to be related to the diminished autophagic activity [223]. Emerging studies have also suggested that ATG5 can promote chemoresistance in different types of cancers [224,225]. Indeed, an increased epirubicin response is observed in both anthracycline-sensitive and -resistant TNBC cells upon knockdown of ATG5 [226]. Combination of autophagy inhibitor and anthracycline has the potential to combat chemoresistance in TNBC (Figure 4) [226].

***Paclitaxel***—As one of the most widely used chemotherapies in metastatic breast cancer, taxane inhibits microtubule depolarization, thus arresting cells at prometaphase and resulting in cell death [227,228,229]. A recent study reported that APRIL, a proliferation-inducing ligand, is related to chemoresistance in TNBC cells [230]; its inhibition sensitizes paclitaxel-resistant TNBC to treatment, whereas overexpression of APRIL promotes chemoresistance [230]. APRIL modulates AKT-mTOR activity [230] to induce autophagy leading to chemoresistance. Indeed, autophagy inhibitors sensitize breast cancer cells to paclitaxel treatment, suggesting that APRIL stimulates the development of chemoresistance by activating autophagy [230]. Interestingly, miR-18a also promotes paclitaxel resistance in TNBC cells by hindering paclitaxel-induced apoptosis and suppressing the expression of Dicer, which is a processer for miRNA [231,232]. DICER promotes autophagy by inhibiting the activation of the PI3K/AKT/mTOR pathway and thus leads to cisplatin resistance non-small cell lung cancer [233]. It is, however, unclear how DICER impacts autophagy in breast cancer paclitaxel resistance. The role of miR-18a and increased autophagy is corroborated by another study that shows how paclitaxel-resistant MDAMB231 cells have higher basal levels of both miR-18a and autophagic activity compared with parental cells [234]. Mechanistically, miR-18a suppresses the mTOR signaling pathway, which in turn leads to an elevated autophagy activity and thus contributes to paclitaxel resistance [234] Interestingly, administration of an autophagy inhibitor yielded a similar, yet stronger effect compared with miR-18a inhibition in paclitaxel-resistant TNBC cells [234]. Even though the mechanistic story of how autophagy contributes to paclitaxel resistance is unclear, it is evident that autophagy is beneficial to chemoresistance in TNBC (Figure 4). 

***Platinum Agents***—The use of platinum agents in treating breast cancer can be dated back to the 1970s [227]. Carboplatin and cisplatin are both platinum agents that function by interfering with DNA strand separation, thus inducing cell death in the middle of DNA replication and transcription [235,236,237]. It is observed that co-administration of carboplatin and autophagy inhibitors results in a drastic reduction in TNBC tumors in vivo [115]. Although there is no other research that directly uncovers the relationship between autophagy and resistance to platinum agents in breast cancer, it has been reported that autophagy is one of the big contributors to the resistance to platinum agents [238]. Upon administration of platinum agents, increased autophagy is reported in esophageal cancer [239], colon cancer [240], liver cancer [241], and neuroblastoma [242]. Accordingly, it has been demonstrated that inhibition of autophagy can reduce platinum resistance in endometrial cancer [243] and ovarian cancer [244].

### 3.5. Autophagy and Immunotherapy—Response and Resistance

**Figure 5:**
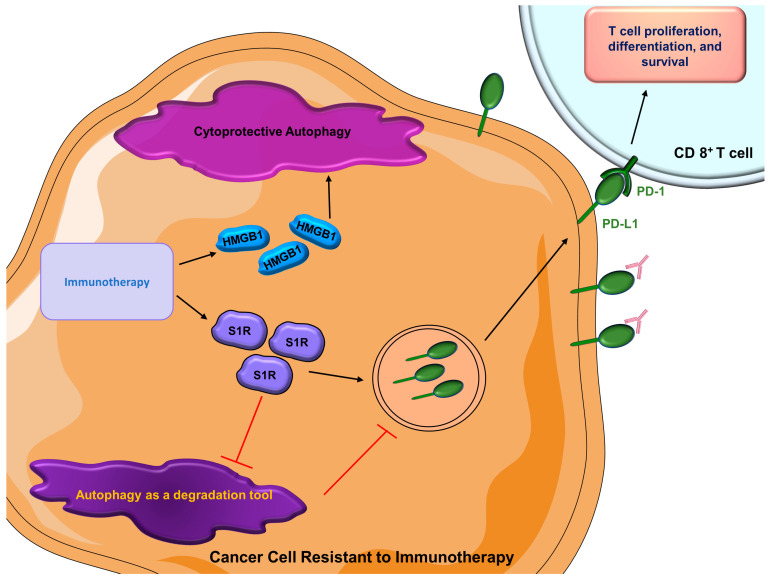
**Roles of autophagy in cancer cells resistant to immunotherapy.** Besides the activation of cytoprotective autophagy, cancer cells’ acquired immunotherapy resistance inhibits degradatory autophagy, thus increasing the amount of surface PD-L1 to interfere with the efficacy of immunotherapy. (HMGB1 = high mobility group box 1; PD-1 = programmed cell death protein 1; PD-L1 = programmed death-ligand 1; S1R = Sigma 1 receptor).

In recent decades, immunotherapy consisting of monoclonal antibodies (anti-PD-1 or anti-PD-L1) has been widely used in breast cancer patients [245,246]. Sigma 1 receptor (S1R) is a unique drug-binding site that is commonly expressed in malignant breast epithelial cells and breast cancer cells [247]. Of interest, inhibition of S1R promotes autophagic flux, resulting in reduced levels of cell surface PD-L1, which succumbs to autophagic degradation [248]. Inversely, treatment with an S1R activator leads to an elevated expression of cell surface PD-L1. This phenomenon is consistent in both TNBC cells and prostate cancer cells [248]. The role of autophagy, in this case, is neither cytoprotective nor cytotoxic; instead, it solely functions as a degradation tool. When S1R is expressed, autophagic flux is inhibited; therefore, PD-L1 is overexpressed on the cancer cell surface, modulating the efficacy of immunotherapy [249]. As aforementioned, HMGB1 may promote autophagy in breast cancer, thus contributing to therapeutic resistance [219]. As a multifunctional redox-sensitive protein, it participates in both intracellular processes such as autophagy, chromatin remodeling, and regulation of transcription [250,251,252,253] and also has roles in extracellular processes such as the regulation of autoimmunity and inflammation [254,255,256]. Recently, a role for HMGB1 blockage in breast cancer and immunotherapy has been uncovered [257]. In immunocompetent mice, co-administration of HMGB1 inhibitor and anti-PD-1 immunotherapy results in a substantial reduction of tumor growth compared with mice who received anti-PD-1 therapy alone [257], suggesting that combination treatment of immunotherapy and an HMGB1 inhibitor can be an optimal strategy. Similarly, autophagy inhibitors combined with immunotherapy lead to an enhanced therapeutic response in pancreatic cancer [258]. There are a couple of reasons that may explain the synergistic effect of autophagy inhibition alongside immunotherapy. Inhibition of autophagy may increase the amount of surface MHC-I on dendritic cells, which strengthens the immune response mediated by CD8^+^ T cells [259]. Additionally, suppression of autophagy can also directly augment the tumor-suppressing ability of CD8^+^ T cells [258,260]. At this point, very limited studies have investigated how autophagy contributes to resistance to immunotherapy in breast cancer. As immunotherapy becomes more prominent, autophagy can be a worthy topic of exploration to increase therapeutic efficacy (Figure 5).

## 4. Inhibition of Autophagy Using Various Inhibitors May Result in Improved Therapeutic Outcomes

### 4.1. Hydroxychloroquine (HCQ)

Hydroxychloroquine is a weak base that inhibits autophagy by accumulating in the lysosomal compartment and thereby inhibiting the formation of autolysosomes [261]. Many preclinical studies that utilize autophagy inhibitors indicate that HCQ can lead to cancer cell death alone or can strengthen the efficacy of various chemotherapies and targeted therapies [262,263,264]. Some clinical trials that attempted to investigate the combination of HCQ and other therapeutics such as Ixabepilone (NCT00765765) or hormonal therapy (NCT02414776) have been terminated due to slow accrual and the departure of PI, respectively, whereas another study that wished to examine the combination of Gedatolisib (NCT03400254) has been withdrawn due to a business decision. HCQ has also been associated with irreversible retinal toxicity with long-term use [265]. However, there are four actively recruiting clinical trials that aim to explore the benefits of HCQ in combination with other therapies in breast cancer, as stated in Table 1.

### 4.2. Chloroquine (CQ)

Compared with HCQ, CQ is less popular in the clinical setting due to ocular toxicity [266]. However, a clinical study explored the efficacy of oral CQ in breast ductal carcinoma in situ (DCIS) (NCT01023477). Upon the completion of this trial, many of the patients had a reduction in autophagy activity and PCNA proliferation index in DCIS lesions [267], implying a decreased rate of invasion and metastasis [268]. Among 12 patients in this clinical trial, 7 of them indicated a shrinkage of the DCIS lesion. Compared with untreated controls, there was an increase in tumor-infiltrating macrophages in the DCIS ducts [267]. Collectively, these results suggested that oral chloroquine led to a significant reduction in DCIS progression along with increased migration of immune cells into the duct [267]. On the other hand, the outcome from a recent clinical trial indicated that chloroquine combined with taxane and related chemotherapy resulted in a 45.16% objective response rate (ORR) [269].

### 4.3. Parthenolide

Parthenolide (PTL) is a sesquiterpene lactone isolated from feverfew [270]. In vitro studies report that PTL hinders proliferation while eliciting apoptosis in breast cancer, cholangiocarcinoma, colorectal cancer, and hepatoma pancreatic cancer [271,272,273]. PTL-induced apoptosis is related to NF-kB inhibition, mitochondrial dysfunction, and an increase in ROS [274,275,276,277,278]. However, treatment with PTL leads to an elevation of autophagic activity in breast cancer cells [279]. Even though PTL increases autophagic activity, autophagy inhibition on top of PTL treatment promotes apoptosis, which proves that PTL-induced autophagy is an attempt to overcome the chemical stress brought by PTL [279]. Hence, PTL supplementation along with autophagy inhibition may suppress the progression of breast cancer.

### 4.4. Honokiol

Honokiol (HNK) is a lignan extracted from Magnolia, which is a common eastern herbal medicine with a long history [280,281]. It has been reported that HNK treatment leads to significant growth reduction and apoptosis in several subtypes of breast cancer [282,283]. Similar to PTL, HNK is a substance extracted from plants; it also induces autophagy in breast cancer as an attempt to subside the stress brought by HNK [284]. The combined treatment with autophagy inhibitors and HNK results in a substantial reduction in breast tumorigenesis and lung metastasis [284].

### 4.5. Withaferin A

Ashwagandha is one of the most wildly used ancient medicine in India [285]. Withaferin A (WFA) is one of the bioactive extracts derived from Ashwagandha, and its anti-neoplastic tendency has been well documented [286,287]. WFA can stimulate autophagy [288], possibly owing to its activation effect on the AMPK pathway [289]. Even though the treatment of WFA activates [286] autophagy in breast cancer cells, it also leads to hyperacetylation of tubulin, thus hindering the fusion of lysosomes and autophagosomes, which results in the build-up of autophagosomes that confer cellular toxicity [288,290]. In addition, treatment with WFA also interferes with Unfolded Protein System (UPS)-mediated proteolysis, which potentiates proteotoxicity and results in cell death [288]. Consistently, our group has shown that WFA is a lysosomal inhibitor [289]. Further, as a lysosomal inhibitor, WFA hinders proteolytic lysosomal activity, causing a deficiency of substrates to feed the citric acid cycle, a vital process of aerobic respiration, resulting in growth inhibition in breast cancer cells [199,289]. These studies imply that WFA inhibits the growth of breast cancer cells and manipulates various aspects of the autophagic pathway. Additional preclinical and clinical studies are required to assess the potential of WFA as a modulator of autophagy that can be beneficial for improving the efficacy of standard therapies for breast cancer.

### 4.6. Toosendanin

Melia toosendan Sieb et Zucc was used as an anthelmintic vermifuge in ancient China [291,292]. The antitumor effect of toosendanin (TSN), a triterpenoid derivative from the bark of Melia roosendan Sieb et Zucc [291,292], has been investigated in recent years. In breast cancer, TSN has the leverage to counter adriamycin resistance in vitro and in vivo via inhibition of the PI2K/Akt signaling pathway and downregulation of ABCB1 [293]. Recently, a study reported that TSN can act as a potent autophagy inhibitor to reverse irinotecan resistance [294]. Unlike widely used autophagy inhibitors such as CQ and HCQ, which disrupt the fusion of lysosome and autophagosome, TSN impairs autophagy by elevating the pH of lysosomes in TNBC cells. Importantly, the effective dose of CQ in vitro is 30 times more than TSN [294], indicating that TSN is a more potent autophagy inhibitor than CQ. To determine whether TSN is a good additive to current treatments of breast cancer, additional preclinical research is needed.

## 5. Conclusions and Future Perspectives

Based on the functional outcomes, autophagy can be cytoprotective (enhances cell survival), cytostatic (leads to growth arrest), cytotoxic (causes cell death), or nonprotective (no impact on cell death or cell survival). However, in terms of interfering with drug efficacy, it is mostly considered cytoprotective. In a bidirectional manner, several drugs modulate the autophagic process and various autophagy-associated proteins directly or indirectly modulate key signaling pathways, miRs and lncRNAs to impact therapeutic efficacy. In last few years, several new targeted therapies or new drug combinations have been put forth especially for metastatic breast cancer, and it is intriguing to note that cancer cells elicit the autophagic response to evade therapy, promote stemness, and induce dormancy in response to many therapeutic strategies. Although, there is ample preclinical evidence to support the strategies combining autophagy inhibitors with anti-cancer drugs and underlying mechanisms have also been explored, clinical studies are few and far between. A major caveat with most autophagy inhibitors is the lack of selectivity towards cancer cells, which ensues overall toxicity since autophagy is important for maintaining homeostasis. Several endeavors are currently underway to develop novel compounds to selectively target autophagy in cancer cells. Focusing on ATG12–ATG3 interaction, a high throughput screen identified a lead compound that selectively inhibits growth in autophagy-addicted cancer cells [295]. A hydrocarbon-stapled peptide directly targeting ATG5–ATG16L1 interaction presents another new strategy for autophagy inhibition [296]. Although discovering and developing new compounds for efficient and selective autophagy inhibition in cancer cells is important, selective delivery of existing autophagy inhibitors is also important. Nanophotosensitizers loaded with chloroquine show increased tumor accumulation and can be sensitized with photodynamic therapy to yield autophagy inhibition [297]. In an interesting strategy, a fasting mimicking diet (FMD) is used to enhance autophagy in cancer cells that are selectively targeted with insulin-like growth factor 2 receptor (IGF2R)-targeted liposomes containing hydroxychloroquine (iLipo-H) [298]. Additional well-designed pre-clinical and clinical studies are required to examine the benefits of adding autophagy inhibitors in combination regimens. Moreover, new autophagy inhibitors with lower toxicity, a better safety profile, and higher efficacy need to be developed to propel this field forward and reap the benefits of preclinical findings.

## Figures and Tables

**Table 1:** List of all clinical trials of autophagy inhibitors in breast cancer.

Phase	Clinical Trial	Treatment	Identifier
*Unknown or Terminated or Withdrawn or Completed*			
2	Autophagy Inhibition Using Hydrochloroquine in Breast Cancer Patients: a Pilot Study	Hydroxychloroquine	NCT01292408 (Unknown status) 2012
1/2	Phase I/II Study of Ixabepilone in Combination With the Autophagy Inhibitor Hydroxychloroquine for the Treatment of Patients with Metastatic Breast Cancer	Ixabepilone and Hydroxychloroquine	NCT00765765 (Terminated) 2013
2	Phase Ib/II Study of Hydroxychloroquine in Metastatic ER-Positive Breast Cancer Progressing on Hormonal Therapy	Hydroxychloroquine in combination with hormonal therapy	NCT02414776 (Terminated) 2015
1/2	Preventing Invasive Breast Neoplasia with Chloroquine (PINC) Trial	High or low dose of Chloroquine	NCT01023477 (Completed) 2016
2	A Phase 2 Randomized, Double-blind, Window of Opportunity Trial Evaluating Trial Clinical and Correlative Effects of Chloroquine as a Novel Therapeutic Strategy in Breast Cancer	Chloroquine and placebo	NCT02333890 (Unknown) 2016
1/2	A Phase Ib/II Trial of Gedatolisib, Hydroxychloroquine or the Combination for Prevention of Recurrent Breast Cancer (“GLACIER”)	Gedatolisib or Hydroxychloroquine alone or in combination	NCT03400254 (Withdrawn) 2020
2	Phase II Study of The Efficacy and Safety of Chloroquine (C) in CombinAtion With Taxane or taxane-like (T) Chemo Agents in The Treatment of Patients With Advanced or Metastatic Breast Cancer Who Have Failed Anthracycline Chemo Base Therapy	Chloroquine with taxane or taxane-like chemotherapy	NCT01446016 (Completed) 2022
*Recruiting or Active*			
1	Hydroxychloroquine (HCQ) in Combination with Abemaciclib and Endocrine Therapy in HR^+^/Her2- Advanced Breast Cancer After a Lead in Dose Escalation Cohort of HCQ and Abemaciclib in Advanced Solid Tumors	Abemaciclib with alternative dose of HCQ or Abemaciclib with both HCQ and endocrine therapy	NCT04316169 (Recruiting) 2028
2	A Phase II Pilot Trial of ABmacocliB or Abemaciclin and HydroxYchloroquine to Target Minimal Residual Disease in Brease Cancer Patients	Abemaciclib with or without Hydroxychloroquine	NCT04523857 (Recruiting) 2028
2	A Phase II Trial of Avelumab or Hydrochloroquine With or Without Palbociclib to Eliminate Dormant Breast Cancer (PALAVY)	HCQ alone, or Avelumab alone, or Avelumab with Palbociclib, or HCQ with Palbociclib	NCT04841148 (Recruiting) 2028
1/2	Phase I/II Safety and Efficacy Study of Autophagy Inhibition With Hydroxychloroquine to Augment the Antiproliferative and Biological Effects of Pre-Operative Palbociclib Plus Letrozole for Estrogen Receptor-Positive and HER2-Negative Breast Cancer	Hydroxycholoroquine, Letrozole, and Palbociclib	NCT03774472 (Active) 2024
2	CLEVER Pilot Trial: A Phase II Pilot Trial of HydroxyChLoroquine, EVErolimus or the Combination for Prevention of Recurrent Breast Cancer	Hydroxychloroquine alone, or Everolimus alone, or the combination of hydroxychloroquine and Everolimus	NCT03032406 (Recruiting) 2025
